# Texas Epileptic Asylum—Dr. Worsham’s Report

**Published:** 1899-08

**Authors:** B. M. Worsham

**Affiliations:** Austin, Texas


### Texas Epileptic Asylum—Dr. Worsham's Report.

AUSTIN, TEXAS, July 3, 1899.

*To His Excellency, Joseph D. Sayers, Governor of Texas.*

DEAR SIR: In compliance with your instructions, I visited the institutions for the care and treatment of epileptics in New York, Massachusetts and Pennsylvania, carefully studied character and arrangement of the buildings, together with the system of management and treatment of the patients.

The plan of colonizing epileptics is comparatively new in this country. Ohio and the three States above named are the only States in America that have adopted this plan and started institutions for the exclusive care of this class of patients. The institution of New York (Craig colony) is the oldest and largest of the three visited, and it has only been opened four years.

In establishing these institutions the principal object has been to separate this class from the insane, and furnish them healthful employment. It is now a well established fact that even the insane do much better and their chances for recovery are largely increased, when employment is at all practicable. This is especially true with the epileptic class, for there are times in the history of most cases when the epileptic seizures are temporarily suspended and for days the patient is practically sane.

The superintendent of the Craig colony said that employment, in his opinion, was the most important factor in the proper care and treatment of epileptics; that if he were forced to adopt only one means of treatment to the exclusion of all others, he would unhesitatingly say employment. With that object in view the founders of Craig colony located it in the most fertile section of New York, and secured a large tract of land, nearly 2000 acres. In planning the arrangement of the buildings and grounds, a complete preliminary drawing or plot was made, dividing the property into sections to be used for farming, gardening, orchard, pasture, etc., and showing the exact location of each building to be erected until the institution is supposed to be complete. This, in my opinion, is a very important feature to be considered in starting a new institution, for the first appropriation made is always sufficient only to start with, and no institution is complete until it has had years of growth. By having a definite plan to go by, whenever new additions are to be made, insures a uniform arrangement throughout.

In most of our State institutions nothing of that sort has been done at the beginning, and as a result we have buildings out of harmony with each other, badly located and improperly ventilated. The modern colony plan consists in erecting numerous inexpensive cottages in groups over a large section of land; one cottage to accommodate from fifteen to thirty patients, and each cottage, when occupied, makes a complete household within itself. This makes it possible to segregate the different types and classes of patients and congregate in households the cases of similar grade and mental capacity, so that each class can receive the care and treatment that their conditions demand.

Industrial groups of buildings, one for men and one for women, are started at the very beginning of the institution, and additions are made to these departments from year to year, to meet the demands of the growth of the institution, thus affording opportunity to furnish diversified employment. Aside from the benefit to the patient derived from healthful employment, the economic value of their labor is of the greatest importance to the State.

At Craig colony every variety of farming and gardening is carried on successfully, and a dairy large enough to meet the demands, a hennery, a piggery and a herd of sheep all add materially to the support of the colony; in fact, most everything that is consumed in the way of food is produced on the place by the labor of the patients.

The records of the institution show that it has been nearly forty-five per cent. self-sustaining this year, and as the institution grows and develops a better showing will be made.

The institution in Massachusetts is practically just starting, and as yet does not represent many of the true colony features. It is located on a small tract of land and the first buildings erected are intended simply for the custodial care of the insane class of epileptics. The plan for future enlargement, however, embraces all of the features of a modern colony with separate groups of cottages, industrial buildings, etc.

The one at Oakburn, Penn., is a very small institution, and is owned and operated by a private corporation. The State, however, made a small appropriation for the past two years to pay for the care and treatment of a very limited number of patients at that place. The last Legislature refused to make such an appropriation for the next two years, and therefore the State care of the patients in that institution has ceased.

The State or public care of the defective and dependent classes is a great and growing burden upon the people in every civilized country; therefore, it is important that in beginning a new institution everything should be considered that might better the condition or increase the chances of the patient's recovery, and at the same time lessen the expense of maintenance and make the institution as near self-sustaining as possible.

In arranging the plans for the proposed colony at Abilene I would recommend that plan adopted and in operation in the State of New York, taking advantage of the experience gained by the splendid management of that institution. Of course, the plans of the buildings will have to be changed to suit the Southern climate. A complete map or landscape drawing should be made of the property where the colony is to be located, showing the exact location of each building, walk, driveway, etc., to be adopted as a guide in all future buildings and development of the institution until it is completed.

When the institution is ready for the admission of patients, I would most strenuously advise against the idea of unloading the insane epileptics now confined in the three insane asylums on the new institution, for nearly all of them have long since reached the stage of complete dementia, and those who have not reached this stage are violent, destructive and very untidy.

With such a population it would be impossible to start off the colony properly, and all the possibilities for farming, gardening and other industries would be at an end. Allow the insane epileptics now confined in the insane asylums to remain where they are, and start the new institution with fresh recruits, for the demands over the State are now great enough to take all of the room that the present appropriation will provide.

I have original plans and drawings of the institutions in New York and Massachusetts, and when you have appointed the architect for the proposed institution at Abilene I will furnish him with the plans and drawings, together with such ideas and notes gathered on my trip of inspection.

Thanking you for the honor conferred on me by sending me on this important mission, and hoping to be of further service in this matter, I am,

Very respectfully,

B. M. WORSHAM.

